# Effect of masticatory stimulation on the quantity and quality of saliva and the salivary metabolomic profile

**DOI:** 10.1371/journal.pone.0183109

**Published:** 2017-08-15

**Authors:** Nobuyuki Okuma, Makiko Saita, Noriyuki Hoshi, Tomoyoshi Soga, Masaru Tomita, Masahiro Sugimoto, Katsuhiko Kimoto

**Affiliations:** 1 Department of Oral Interdisciplinary Medicine, Graduate School of Dentistry, Kanagawa Dental University, Kanagawa, Japan; 2 Institute for Advanced Biosciences, Keio University, Yamagata, Japan; 3 Division of Environmental Pathology, Department of Oral Science, Graduate School of Dentistry, Kanagawa Dental University, Kanagawa, Japan; 4 Health Promotion and Preemptive Medicine, Research and Development Center for Minimally Invasive Therapies, Tokyo Medical University, Shinjuku, Tokyo, Japan; Cleveland Clinic, UNITED STATES

## Abstract

**Background:**

This study characterized the changes in quality and quantity of saliva, and changes in the salivary metabolomic profile, to understand the effects of masticatory stimulation.

**Methods:**

Stimulated and unstimulated saliva samples were collected from 55 subjects and salivary hydrophilic metabolites were comprehensively quantified using capillary electrophoresis-time-of-flight mass spectrometry.

**Results:**

In total, 137 metabolites were identified and quantified. The concentrations of 44 metabolites in stimulated saliva were significantly higher than those in unstimulated saliva. Pathway analysis identified the upregulation of the urea cycle and synthesis and degradation pathways of glycine, serine, cysteine and threonine in stimulated saliva. A principal component analysis revealed that the effect of masticatory stimulation on salivary metabolomic profiles was less dependent on sample population sex, age, and smoking. The concentrations of only 1 metabolite in unstimulated saliva, and of 3 metabolites stimulated saliva, showed significant correlation with salivary secretion volume, indicating that the salivary metabolomic profile and salivary secretion volume were independent factors.

**Conclusions:**

Masticatory stimulation affected not only salivary secretion volume, but also metabolite concentration patterns. A low correlation between the secretion volume and these patterns supports the conclusion that the salivary metabolomic profile may be a new indicator to characterize masticatory stimulation.

## Introduction

Saliva is secreted from salivary glands, but also contains gingival crevicular fluid, serum from the mucosa, epithelial cells, and immune cells that are not derived from the salivary glands [1–2]. Saliva is an easily accessible biofluid that contains hundreds of components, possibly providing both oral and systematic information. Accumulated evidence has shown that the systematic cascade of appropriate salivary flow is indicative of systemic health, because the development, function, and differentiation of salivary gland cells affects the general health of a whole organism [3].

Occlusal masticatory stimulation plays an important role in the quality of life of patients. Saliva is crucial in mastication, and its volume and chemical properties affect immune functions and maintenance of health [4–5]. Occlusal masticatory stimulation increases both the rate of salivary secretion and volume [6], affecting salivary pH and enzyme function [7–8]. These changes are also expected to affect the intraoral environment, and thus occlusal masticatory stimulation has a considerable effect on various salivary chemical components.

Comprehensive molecular profiling, for example, omics studies of mRNA and proteins, have been intensively performed to better understand new biological findings and biomarker discoveries [9–10]. Small organic compounds (<1500 Da), named metabolites, have also been revealed to be informative salivary phenotypes with respect to various diseases, including periodontal diseases, leukoplakia, and cancers [11–14]. Comparisons of metabolomic profiles of stimulated and unstimulated saliva have been reported, and those using nuclear magnetic resonance (NMR) for a few high concentration metabolites [15], and gas chromatography (GS) for fatty acids [16].

In this study, we profiled hundreds of hydrophilic metabolites using capillary electrophoresis-mass spectrometry (CE-MS) [17], to understand the effect of occlusal masticatory stimulation on the wider metabolomic phenotype of saliva. We analyzed the correlation between salivary secretion volume and metabolomic profile.

## Materials and methods

### Saliva collection

This study was approved by the Ethics Committee of Kanagawa Dental University (no. 243). Volunteers to provide saliva were recruited at Kanagawa Dental University. Written, informed consent was obtained from volunteers who agreed to serve as saliva donors. Unstimulated saliva and stimulated saliva were collected from 55 subjects using the spitting method and gum test (GC, Tokyo, Japan) [18]. Based on Takeda et al. report [15], we elected to perform measurements in 55 samples. Subject characteristics are summarized in Table 1. The present study compared saliva samples collected by the same examiner from subjects at rest and during stimulation by mastication, during the same time period. Although methods for collection of parotid gland saliva and palatine gland saliva have been reported [19–20], it is difficult to collect samples during mastication. In the present study, we focused on the assessment of metabolites from whole saliva as a practical collection method for human subjects. Saliva was collected from each subject at the same time of day (16:00), and after fasting for at least 2 h after lunch. First, unstimulated saliva was collected over a period of 15 min. Subsequently, stimulated saliva was collected using the gum test for a period of 10 min. Gustatory stimuli are reported to cause changes in saliva volume [21]. In the present study, we used flavorless, odorless gum to examine stimulation produced by mastication alone. Therefore, the effects of taste and smell are considered to be minimal. During the collection, the sample tubes were kept on ice. Secretion volume was measured during sample collection. The samples were centrifuged at 500 × g (CF-15RX, Hitachi, Tokyo, Japan) for 15 min at 4°C for separation. The supernatant was transferred to a fresh tube and the samples were immediately stored at -80°C.

**Table 1:** Subject characteristics.

Characteristics		n	Ave	Stdev	*P*-Value
Age					
Total		55	28.15	3.82	-
Sex	Male	36	28.22	3.64	0.61
Age	Female	19	28.00	4.23	
Smoking	Yes	9	28.89	4.11	0.51
	No	46	28.00	3.79	
Unstimulated salivary volume (mL)					
Total		55	7.14	2.62	-
Sex	Male	36	7.29	3.05	0.57
	Female	19	6.87	1.57	
Smoking	Yes	9	6.69	0.83	0.36
	No	46	7.23	2.84	
Stimulated salivary volume (mL)					
Total		55	14.33	3.55	-
Sex	Male	36	14.53	3.73	0.72
	Female	19	13.94	3.26	
Smoking	Yes	9	13.88	2.51	0.50
	No	46	14.41	3.39	

Note: *P*-values were calculated using Mann–Whitney test

### Metabolomic analysis

Metabolomic analysis were used in this study as previously described [22]. Frozen saliva was thawed and filtered to remove macromolecules by centrifugation through a 5 kDa cut-off filter (Pall, Tokyo, Japan) at 9,100 × g for at least 2.5 h at 40°C. Five microliters of Milli-Q water (Millipore, Bedford, MS) (containing 2 mmol/L each of methionine sulfone, 2-[N-morpholino]-ethanesulfonic acid, D-camphor-10-sulfonic acid, 3-aminopyrrolidine, and trimesate) was added to 45 μL of the filtrate and mixed. CE-time-of-flight-MS was used to simultaneously quantify charged metabolites in positive and negative modes.

### Measurement conditions and instrument parameters

Measurement conditions and instrument parameters have been previously described [22], but used in this study with slight modification. Cation analysis was performed using an Agilent CE system (G1600AX), an Agilent G1969A LC/MSD time-of-flight (TOF) system, an Agilent 1200 series isocratic high performance liquid chromatography (HPLC) pump, a G3251A Agilent CE-MS adapter kit, and a G1607A Agilent CE-electrospray ionization (ESI)-MS sprayer kit (Agilent Technologies, Waldbronn, Germany). Anion analysis was performed using an Agilent CE system (G1600AX), an Agilent G1969A LC/MSD TOF system, an Agilent 1200 series isocratic HPLC pump, a G3251A Agilent CE-MS adapter kit, and a G1607A Agilent CE-ESI source-MS sprayer kit (Agilent Technologies, Tokyo, Japan). For the cation and anion analyses, the CE-MS adapter kit included a capillary cassette that facilitates thermostatic control of the capillary. The CE-ESI-MS sprayer kit simplifies coupling of the CE system with the MS system and is equipped with an electrospray source. For system control and data acquisition, Agilent ChemStation software for CE (B.03.02 and B.02.01.SR1) and Agilent MassHunter software for TOF-MS (B.02.00) were used. The original Agilent SST316Ti stainless steel ESI needle was replaced with a passivated SST316Ti stainless steel and platinum needle (passivated with 1% formic acid and a 20% aqueous solution of isopropanol at 80°C for 30 min) for anion analysis.

For cationic metabolite analysis using CE-TOF-MS, sample separation was performed in fused silica capillaries (50 μm inner diameter × 100 cm total length) filled with 1 mol/L formic acid as the running electrolyte. The capillary was flushed before each sample injection with ammonium formate for 5 min, followed by Milli-Q for 5 min, and run buffer for 5 min. Sample solutions (approximately 3 nl) were injected at 50 mbar for 5 s and a voltage of 30 kV was applied. Capillary temperature was maintained at 20°C, and the temperature of the sample tray was kept below 5°C. The sheath liquid, composed of methanol/water (50% v/v) and 0.1 μmol/L hexakis(2,2-difluoroethoxy)phosphazene (Hexakis), was delivered at 10 μL/min. ESI-TOF-MS was conducted in the positive ion mode. The capillary voltage was set at 4 kV and the flow rate of nitrogen gas (heater temperature of 300°C) was set at 7 psig. For TOF-MS, the fragmentor, skimmer and OCT RF voltages were 75, 50 and 125 V, respectively. Automatic recalibration of each acquired spectrum was performed using reference standards (13C isotopic ion of protonated methanol dimer [2MeOH + H]+, 66.0631 m/z) and (protonated Hexakis [M + H]+, 622.0290 m/z). Mass spectra were acquired at a rate of 1.5 cycles over a range of 50 m/z to 1000 m/z.

For anionic metabolite analysis using CE-TOF-MS, a commercially available COSMO(+) capillary (50 μm × 105 cm) (Nacalai Tesque, Kyoto, Japan), chemically coated with a cationic polymer, was used for separation. Ammonium acetate solution (50 mmol/L; pH 8.5) was used as the electrolyte for separation. Before each injection, the capillary was equilibrated for 2 min by flushing with 50 mmol/L acetic acid (pH 3.4) and then with the running electrolyte for 5 min. The sample solution (approximately 30 nl) was injected at 50 mbar for 30 s, and a voltage of 30 kV was applied. Capillary temperature was maintained at 20°C and the sample tray was cooled to below 5°C. An Agilent 1100 series pump equipped with a 1:100 splitter was used to deliver 10 μL/min of 5 mM ammonium acetate in 50% (v/v) methanol/water, containing 0.1 μM Hexakis, to the CE interface. Here, it was used as the sheath liquid surrounding the CE capillary to provide a stable electrical connection between the tip of the capillary and the grounded electrospray needle. ESI-TOF-MS was conducted in the negative ionization mode at a capillary voltage of 3.5 kV. For TOF-MS, the fragmentor, skimmer and OCT RF voltages were set at 100, 50 and 200 V, respectively. The flow rate of the drying nitrogen gas (heater temperature of 300°C) was maintained at 7 psig. Automatic recalibration of each acquired spectrum was performed using reference standards (13C isotopic ion of deprotonated acetic acid dimer [2 CH3COOH-H]-, 120.03841 m/z), and (Hexakis deprotonated acetic acid [M + CH3COOH-H]-, 680.03554 m/z). Exact mass data were acquired at a rate of 1.5 spectra over a range of 50 m/z to 1000 m/z.

### Data analysis

Raw data were analyzed using MasterHands (version 2.17.0.10, Keio University, Yamagata, Japan), which detected all possible peaks, eliminated noise and redundant features, and generated the aligned data matrix [12, 23]. Metabolite identification was conducted by matching the m/z values and the normalized migration times with our standard compounds, and the concentrations of individual metabolites were calculated using both internal and external standards [22].

Principal component analyses (PCA) were conducted to assess the overall diversity in the metabolomic profiles. The first and second PC (PC1 and PC2) were individually analyzed by subject characteristics. Using two-dimensional PCA, Takeda et al. reported that salivary metabolites from samples taken at rest differ from those obtained after stimulation of saliva production by acid [15]. Analogous to these analyses, we assessed the differences between saliva taken at rest and saliva taken during stimulation by mastication. The Wilcoxon matched pairs test, Spearman correlation, and Wilcoxon test were used for the analyses of sampling methods and the other characteristics, respectively. The Kruskal-Wallis test was used to assess the age-dependent differences. *P*-values for metabolites were corrected using a false discovery rate (FDR) to account for multiple testing [24]. Metabolite data showing correlated *P*-values that were less than 0.05 were transferred to pathway analysis using MetaCore (Thomson Reuters Inc., Carlsbad, CA). The geometric mean of fold change (FC) of the concentration of stimulated saliva divided by the concentration of unstimulated saliva was calculated among samples. Maximum FC and half of the minimum FC among the 55 subjects were substituted for the undetected cases in unstimulated and stimulated peaks, respectively.

These data analyses and visualizations were conducted XLStat (version 2014.1.09, XLStat, Paris, France), R-software (2.14.0, R Foundation for Statistical Computing, Vienna, Austria, www-R-project.org), and GraphPad Prism (version 5.0.2 GraphPad Software Inc., San Diego, CA).

## Results

### Overall view of metabolomic profiles

**Fig 1:**
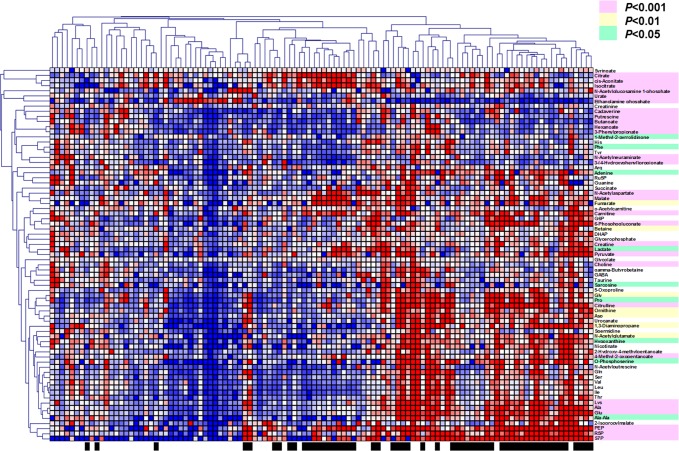
Heat map of salivary metabolite concentrations. Red, white, and blue indicate relatively higher, average and lower concentrations, respectively. Those identified by the black box (bottom of the figure) were from the stimulated saliva samples, and the others were from the unstimulated samples. Clustering was conducted using Pearson correlation. Metabolite names are colored depending on the corrected *P*-values between the 2 groups.

In total, 137 metabolites were identified and quantified. Of these, 76 metabolites were detected at ≥80% concentration among samples in either stimulated or unstimulated saliva samples. Among these, three metabolites showed excessively high average concentrations (>500 μM). Urea, propionate, and 5-aminovalerate were partially saturated and eliminated for the subsequent analysis. The remaining 73 metabolites were visualized in a heat map (Fig 1). The samples were not perfectly clustered into two classes. However, most of the unstimulated saliva samples were aligned to the left side while the stimulated samples were aligned to the right side. Samples that showed similar salivary metabolomic profiles, i.e. metabolite concentration patterns, were aligned closely with each other in this map. Most of the unstimulated saliva samples showed similarity with respect to their profiles, and most of the stimulated saliva samples also showed this tendency. Forty-four metabolites in the stimulated saliva samples showed higher concentrations at significant levels (*P*<0.05, correlated by FDR). For example, amino acids were clustered into three groups: (1) histidine, phenylalanine, tryptophan, and arginine; (2) glycine, proline, asparagine; and (3) glutamine, serine, valine, leucine, isoleucine, threonine, lysine, alanine, and glutamic acid.

**Fig 2:**
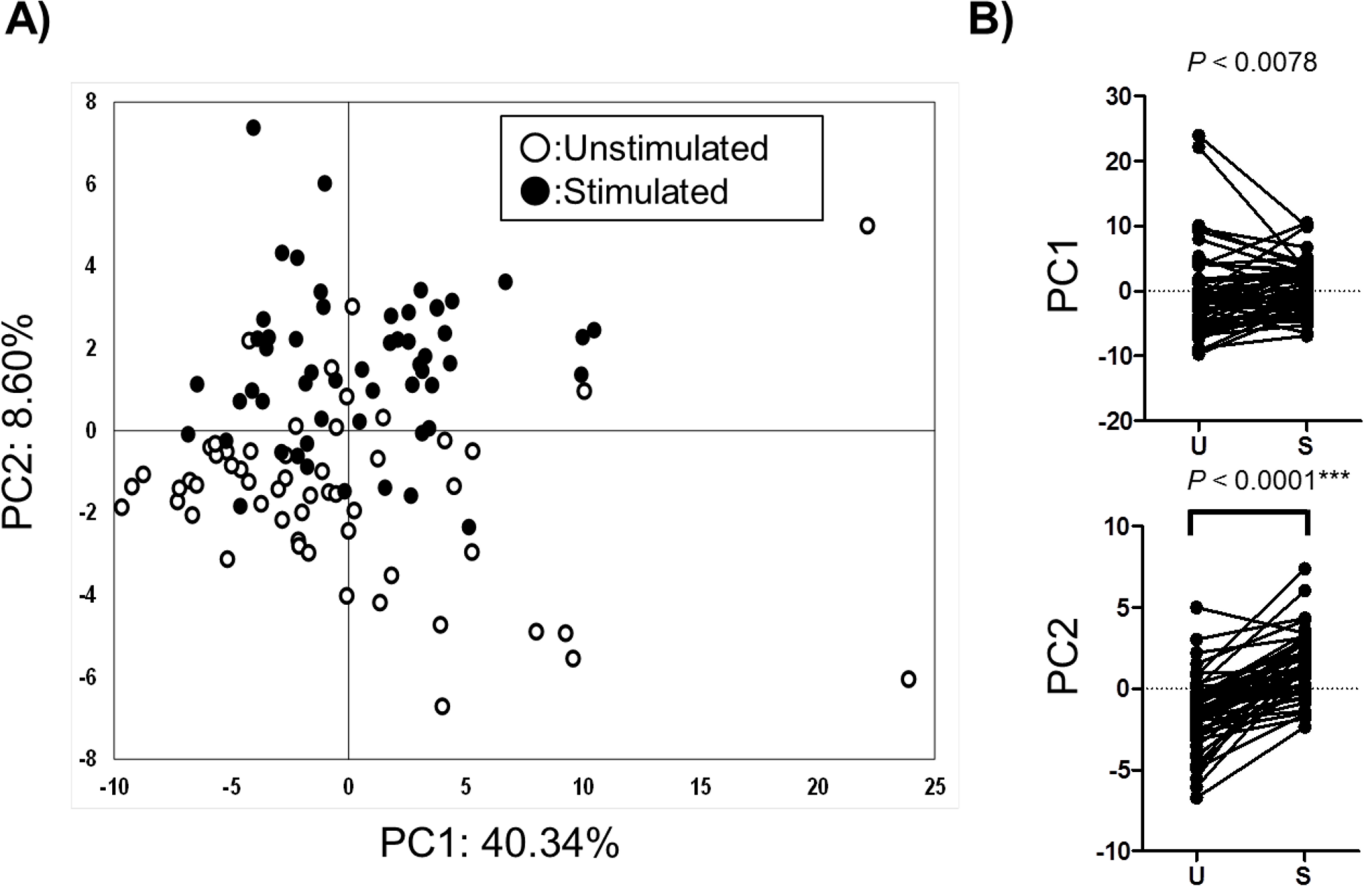
Score plots of principal component analysis (PCA) of salivary metabolomic profiles, where **A)** unstimulated (open) and stimulated (filled); and **B)** line-dot plots of PC1 and PC2. Contribution ratios of PC1 and PC2 are described at the X and Y axis. P-values were calculated using the Wilcoxon matched pairs test.

**Fig 3:**
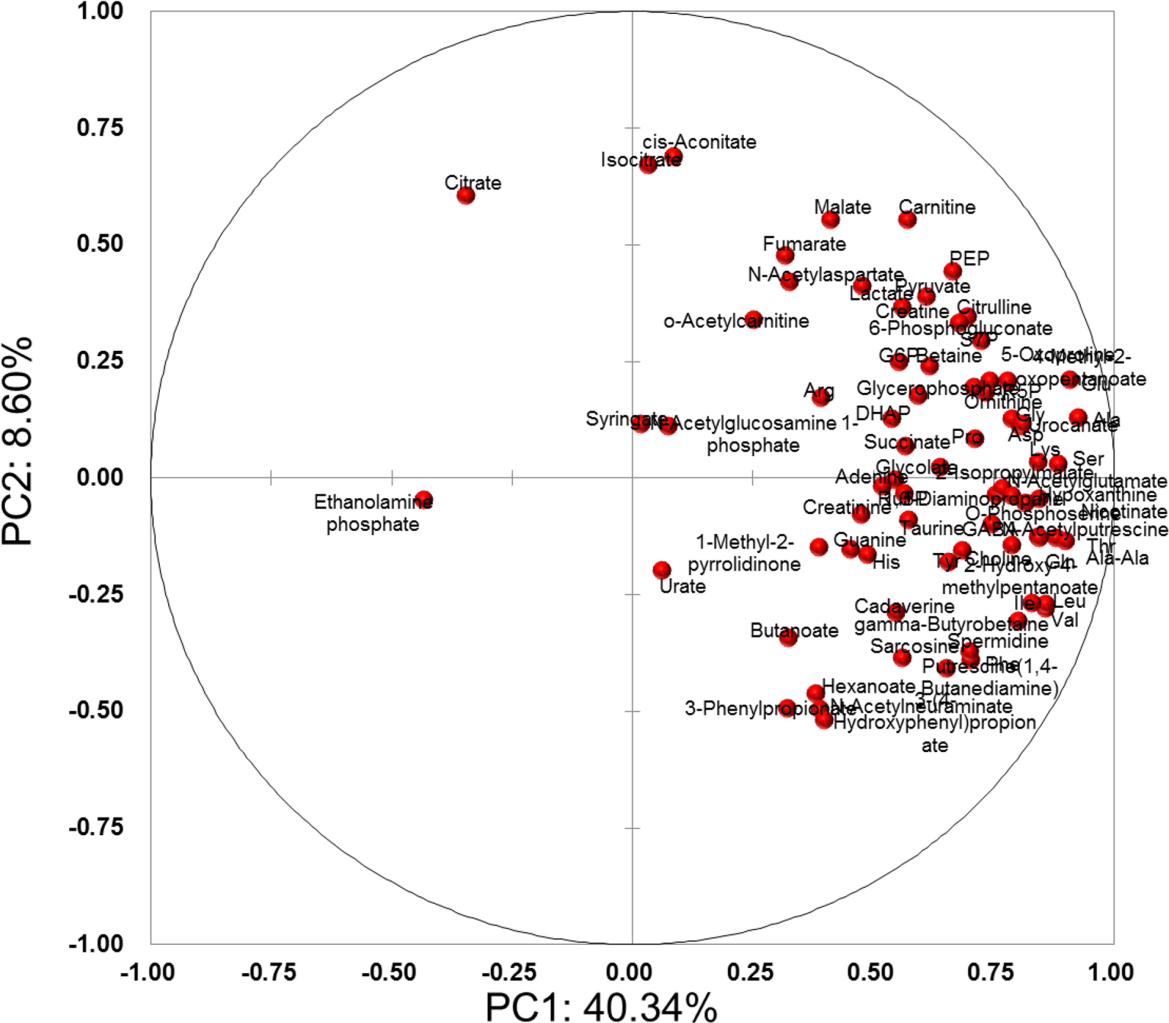
Loading plots of principal component analysis (PCA).

Score plots and loading plots of PCA using the 76 metabolites were visualized (Figs 2A and 3). In the loading plots (Fig 3), all metabolites except for citrate and ethanolamine phosphate were aligned in right side, i.e. PC1>0, indicating that PC1 values positively correlated with overall metabolite concentrations. Meanwhile, metabolites were sparsely distributed in PC2 (Fig 3), indicating that the PC2 values carried the subject’s specific metabolite patterns. Only PC2 showed significant differences (*P*<0.0001, Wilcoxon matched pairs test) in stimulated saliva samples (Fig 2B).

The comparison of these PC values with age (S1 Fig) revealed that PC1 values monotonically increased only in unstimulated saliva samples and at significant levels (*P*<0.0001, Kruskal-Wallis test). This indicates the increase in overall metabolomic concentration with age. In contrast, no other comparisons showed significant changes.

In the comparison of sex (S2 Fig) with PC values, the PC1 values of unstimulated saliva from males were significantly higher than those of females (S2A Fig). In the comparison of matched stimulated and unstimulated saliva samples for each sex, PC1 significantly increased in females (*P =* 0.0016) and PC2 significantly increased in both sexes (*P*<0.0001 for males and *P* = 0.0001 for females) (S2B Fig).

With respect to smoking, both unstimulated and stimulated saliva samples showed no significant differences (S3A Fig). In contrast, a comparison of matched samples in these cases indicated higher values of PC2 in both smoking and non-smoking. (*P* = 0.0039 for males and *P*<0.0001 for females) (S3B Fig).

### Correlation between metabolomic profiles and salivary secretion volumes

**Fig 4:**
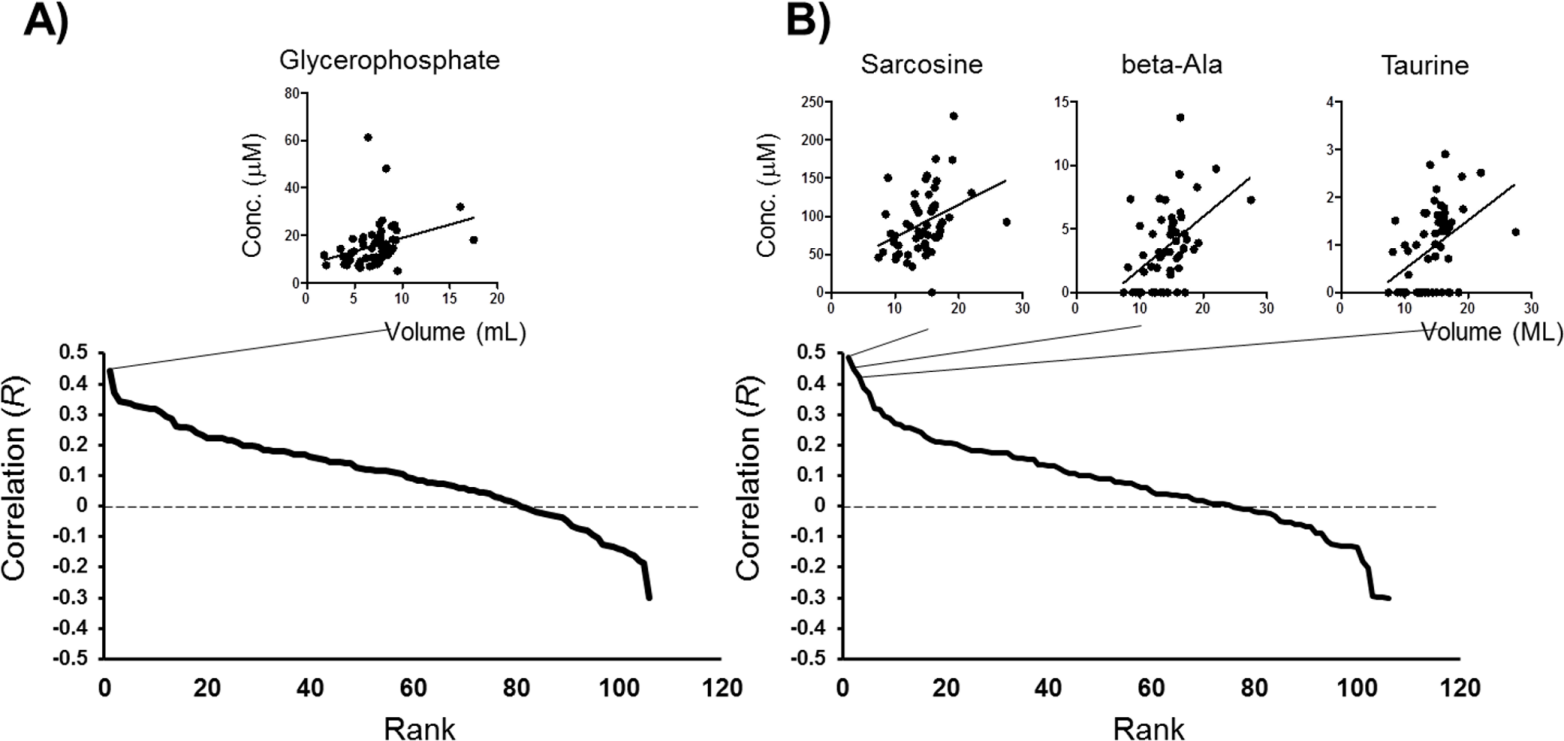
Correlation between metabolite concentration of salivary volume for unstimulated **(A)** and stimulated **(B)** saliva. The relationship between metabolite concentration and salivary volume for the metabolites was significantly correlated (*P*-value<0.05, Spearman ranked correlation), and is visualized in small panels. Regression lines show the overall trends.

Correlation between metabolite concentrations (*R*) and salivary secretion volumes of unstimulated saliva ranged from 0.44 to -0.30, and only glycerophosphate showed a significant correlation *R* = 0.44 (*P* = 0.040, Spearman test) (Fig 4A). With respect to stimulated saliva, correlation values ranged from 0.49 to -0.30, and only sarcosine, beta-alanine, and taurine (*R* = 0.49, 0.45 and 0.42, respectively, and *P* = 0.0090, 0.022, and 0.035, respectively) showed significant correlation (Fig 4B).

### Pathway analysis of metabolomic profiles

**Fig 5:**
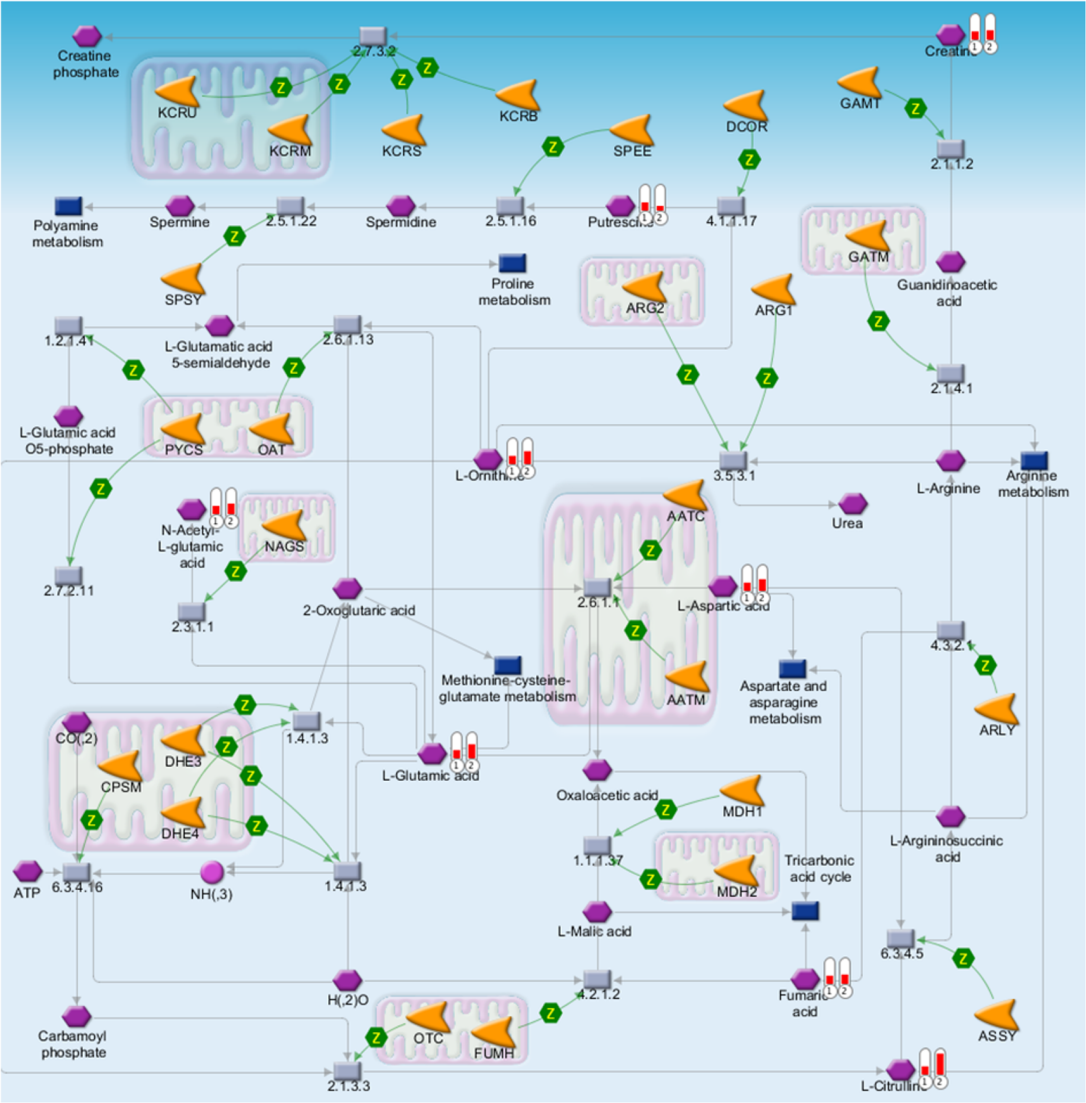
The metabolic pathways that ranked first in the pathway analysis, and using only metabolites, showed significant differences (*P*<0.05, Wilcoxon matched pairs test, corrected by false discovery rate) between unstimulated and stimulated saliva samples. The red bar indicates the average concentrations of unstimulated (left) and stimulated (right) saliva. To clearly visualize the data, these data were divided by the average of unstimulated saliva.

Metabolites belonging to specific pathways were assessed by pathway analysis. Table 2 ranks metabolic pathways showing differences between unstimulated and stimulated saliva. The urea cycle was ranked highest. In this pathway, and with the exception of putrescine (FC = 0.64, corrected *P*-value = 1.5×10^−5^), citrulline (FC = 2.8, corrected *P*-value = 1.9×10^−5^), carnitine (FC = 1.9, corrected *P*-value = 3.2×10^−8^), glutamic acids (FC = 1.9, corrected *P*-value = 1.1×10^−4^), orinitine (FC = 1.6, corrected *P*-value = 0.0099), aspartic acids (FC = 1.5, corrected *P*-value = 0.0072), and *N*-acetylglutamate (FC = 1.4, corrected *P*-value = 0.0061), were elevated (Fig 5). Amino acid metabolism pathways followed with lower ranks, for example glycine, serine and cysteine (2nd), asparatate and asparagine (5th), and alanine, cysteine and methionine (11th). Primary pathways, such as the tricarbonic acid cycle (8th), pentose phosphate pathway (38th), and pyruvate metabolism (44th), were also included.

**Table 2:** Ranking of pathways and related correlation between unstimulated and stimulated saliva samples.

Rank	Pathway	*P*-Value	Correlated *P*-Value
1	Urea cycle	1.1E-09	1.5E-07
2	Glycine, serine, cysteine and threonine metabolism	8.7E-08	3.5E-06
3	Aminoacyl-tRNA biosynthesis in mitochondrion	9.5E-08	3.5E-06
4	Aminoacyl-tRNA biosynthesis in cytoplasm	3.3E-07	8.3E-06
5	Aspartate and asparagine metabolism	1.2E-06	2.3E-05
6	(L)-Arginine metabolism	1.4E-06	2.3E-05
7	Nociception_Pro-nociceptive action of Nociceptin in spinal cord at low doses	1.5E-06	2.3E-05
8	Tricarbonic acid cycle	4.0E-06	5.6E-05
9	Neurophysiological process_Circadian rhythm	7.1E-05	8.3E-04
10	Histidine-glutamate-glutamine metabolism	8.5E-05	9.1E-04
11	L-Alanine, L-cysteine, and L-methionine metabolism	1.4E-04	1.4E-03
12	Glycolysis and gluconeogenesis p.3	1.9E-04	1.6E-03
13	Neurophysiological process_nNOS signaling in neuronal synapses	3.4E-04	2.5E-03
14	Regulation of lipid metabolism_PPAR regulation of lipid metabolism	1.0E-03	7.3E-03
15	Nicotine signaling in cholinergic neurons	1.7E-03	1.2E-02
22	Proline metabolism	1.8E-03	1.2E-02
23	Mitochondrial dysfunction in neurodegenerative diseases	2.8E-03	1.7E-02
24	Glycine links	4.8E-03	2.7E-02
25	Glutamate links	4.8E-03	2.7E-02
26	N-Acylethanolamines, HSRL5-transacylation pathway	7.3E-03	3.9E-02
27	Lysine metabolism	7.5E-03	3.9E-02
29	Regulation of lipid metabolism_Insulin regulation of fatty acid metabolism	8.8E-03	4.2E-02
31	Nicotine signaling in glutamatergic neurons	1.1E-02	4.9E-02
32	Cannabinoid receptor signaling in nicotine addiction	1.1E-02	4.9E-02
33	Beta-alanine metabolism	1.1E-02	4.9E-02
34	Nitrogen metabolism	1.2E-02	5.0E-02
36	IMP biosynthesis	1.6E-02	6.1E-02
38	Pentose phosphate pathway	1.8E-02	6.1E-02
39	Leucine, isoleucine and valine metabolism	1.8E-02	6.1E-02
40	Methionine-cysteine-glutamate metabolism	1.8E-02	6.1E-02
41	Neurophysiological process_PGE2-induced pain processing	1.8E-02	6.1E-02
42	Immune response_IL-13 signaling via JAK-STAT	1.9E-02	6.2E-02
43	Nicotine signaling in dopaminergic neurons, Pt. 1—cell body	2.2E-02	7.1E-02
44	Pyruvate metabolism	2.3E-02	7.1E-02
45	Neurophysiological process_Long-term depression in cerebellum	2.3E-02	7.1E-02
46	Impaired NO signaling in CF airways	2.4E-02	7.1E-02
47	Immune response_IL-13 signaling via PI3K-ERK pathway	2.4E-02	7.1E-02
48	Gamma-aminobutyrate (GABA) biosynthesis and metabolism	2.7E-02	7.7E-02
49	Neurophysiological process_Constitutive and activity-dependent synaptic AMPA receptor delivery	3.2E-02	9.2E-02
50	Glutathione metabolism	3.8E-02	1.0E-01

## Discussion

The quantitative relationship between masticatory stimulation and the composition of saliva was investigated using a metabolome analysis. The correlation between salivary secretion and volume were also analyzed in this study. The mechanism for salivary secretion from acinar cells is well known; the fluid secretion is dependent on the cascade of signals starting from acetylcholine release from parasympathetic nerves, activation of muscarinic M3 receptors, and elevation of cytoplasmic calcium concentrations [25]. Protein secretion is also characterized by the binding of noradrenaline in sympathetic nerves at β1-adrenoceptors, and vasointestinal peptide (VIP) release from parasympathetic nerves to VIP receptors and pituitary adenylate cyclase polypeptide receptors. This leads to the elevation of cyclic AMP, activation of protein kinase A, and induction of protein secretions [25]. A similar mechanism of metabolite secretion is expected. However, reports on metabolite secretion are few, and this study focused on salivary metabolite profiles.

Comparisons of unstimulated and stimulated saliva secretions have been investigated in several reports. Takeda et al. employed proton nuclear magnetic resonance (^1^H NMR) to quantify 18 salivary metabolites from males, and revealed higher concentrations of 9 metabolites, including alanine, glycine, lactate, and taurine, in stimulated saliva [15]. This was consistent with our observations. The volume of stimulated saliva was compared with the volume of unstimulated saliva among sample groups to resolve the postulation of the effect of dilution on the concentration of the metabolites. However, most of the metabolites in stimulated saliva were higher in concentration or almost constant compared with those in the unstimulated saliva. Although Takeda et al. examined stimulation by acid, the objective of the present study was to compare salivary metabolites associated with stimulation by mastication. Therefore, the present study is novel in that flavorless, odorless gum was used to compare salivary metabolites associated with stimulation by mastication. Additionally, whereas Takeda et al. analyzed salivary metabolites using ^1^H NMR spectroscopy, we conducted high-performance analysis with CE-MS [15]. The present study is also original in that it features pathway analysis. We have previously quantified charged metabolites in saliva obtained from healthy young subjects with reasonable oral hygiene (without periodontal disease or gingival inflammation at the least) using the swab and passive drool methods [22]. Both groups of saliva samples were collected by unstimulated methods. However, these salivary metabolomic profiles differed, and probably due to the contact of the swab in the oral cavity which would stimulate the salivary glands despite the low intensity of stimulation. The PCA of these data also showed that the first PC reflected the overall concentration of saliva, which was consistent with the observations in this study.

The first and second PC values were compared with age, sex, and smoking. Interestingly, the PC1 values of unstimulated saliva were clearly dependent on age, despite no significant differences among other comparisons (S1 Fig). Because the PC1 is a factor that characterizes the overall concentration of salivary metabolites (Fig 3), the overall concentration of unstimulated saliva was determined to depend on age. We collected samples from subjects aged 25–39 years, as individuals in this age range are not affected markedly by caries and periodontal disease. In future, it would be interesting to conduct further assessment of individuals representing a wider age range. Unstimulated and stimulated saliva samples were also compared with respect to sex and smoking factors. The change in PC2 was clearer than for PC1 (S2B and S3B Figs), which implies the change in salivary metabolomic characteristics orthogonal to overall concentrations.

Pathway analysis revealed that metabolites changed in many amino acid pathways between unstimulated and stimulated saliva samples (Table 2). These pathways are connected to each other, for example, both the urea cycle (1st) and arginine (6th) are well known as aberrant in inflammation [26], and the methionine-cysteine-glutamate pathway (40th) and glutathione metabolism (50th) are indicators of oxidative stress [27]. The metabolites in these pathways were expected to accumulate in salivary glands. However, we did not perform measurements of the metabolites in salivary glands, but only in saliva samples. This made it difficult to identify the source of metabolite secretion. The use of isotope-labeled metabolites is one way to help identify this source, and is the subject of further study.

There are limitations to be acknowledged. We utilized CE-MS to profile hydrophilic metabolites. Because 99% of the saliva is water, CE-MS is suitable for profiling a wide range of metabolites. However, the profile is not comprehensive, and other less water-soluble chemical compounds, such as lipids, have been confirmed to exist in saliva [28–29]. Interestingly, the metabolites were not correlated with salivary volume. This implies that the metabolomic profiles have potential to be used as indicators, orthogonal to salivary volume, to characterize the salivary grands. Further analyses with a variety of salivary functions are needed.

## Supporting information

S1 Fig Relationships between age and principal component (PC) values in Fig 2. *P*-Values were calculated using the Kruskal-Wallis test.
(TIF)

S2 Fig Relationships between sex and principal component (PC) values in Fig 2. Comparisons between **(A)** sex, and **(B)** sex for unstimulated and stimulated saliva groups. *P*-values were calculated using the **(A)** Mann-Whitney test and Wilcoxon matched pairs test **(B)**, respectively.
(TIF)

S3 Fig Relationships between smoking and principal component (PC) values in Fig 2. Comparisons between **(A)** smoking and non-smoking, and **(B)** comparison of unstimulated and stimulated saliva among subjects with each smoking habit. *P*-values were calculated using **A)** the Mann-Whitney test, and **B)** Wilcoxon matched pairs test.
(TIF)
