# Supplementary figures and images for: Effect of masticatory stimulation on the quantity and quality of saliva and the salivary metabolomic profile
Source: PLoS One. 2017 Aug 15;12(8):e0183109. doi: 10.1371/journal.pone.0183109 (PMC5557591; doi:10.1371/journal.pone.0183109)

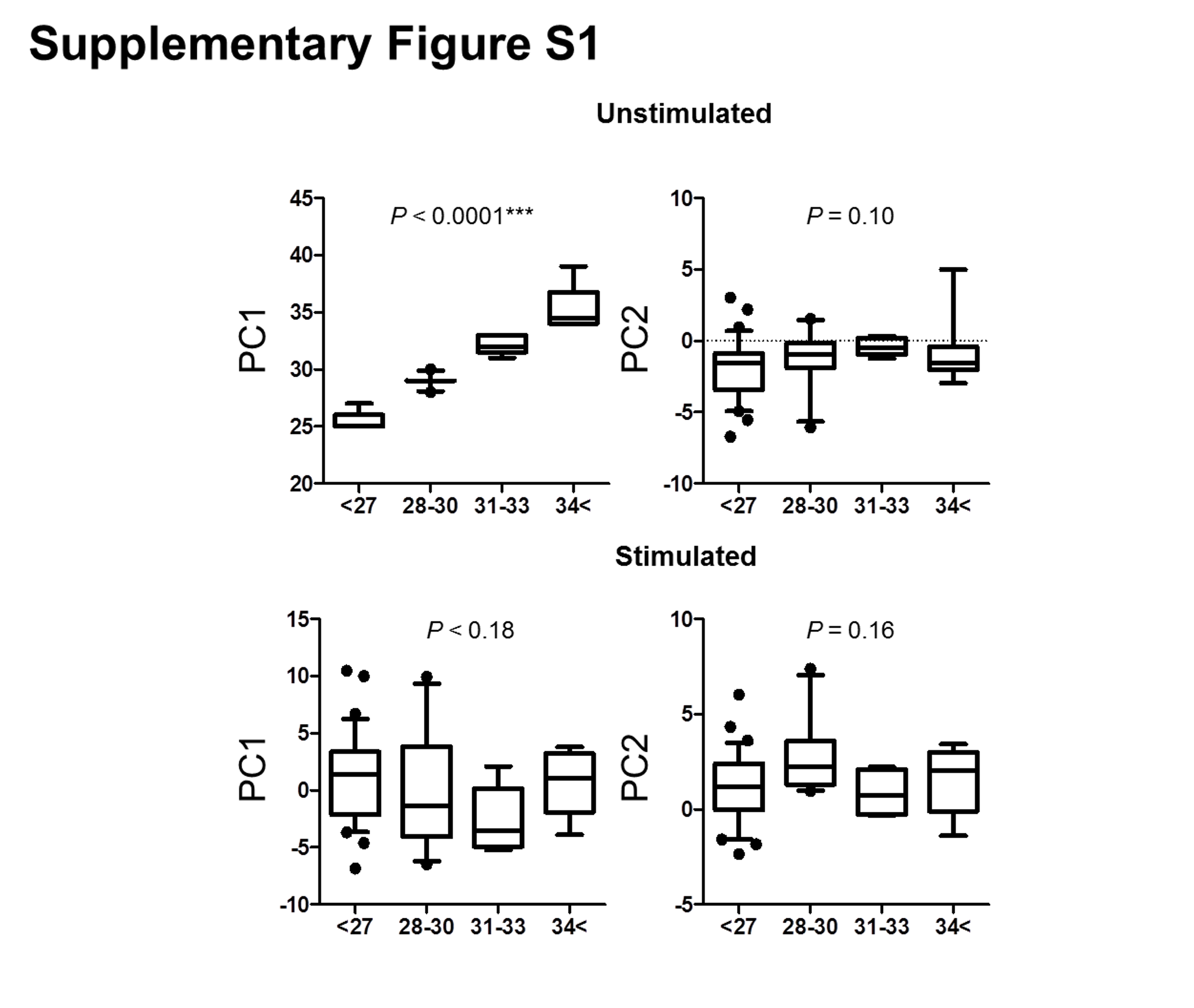

Supplement: S1 Fig — P-Values were calculated using the Kruskal-Wallis test. (TIF) [file pone.0183109.s001.tif]

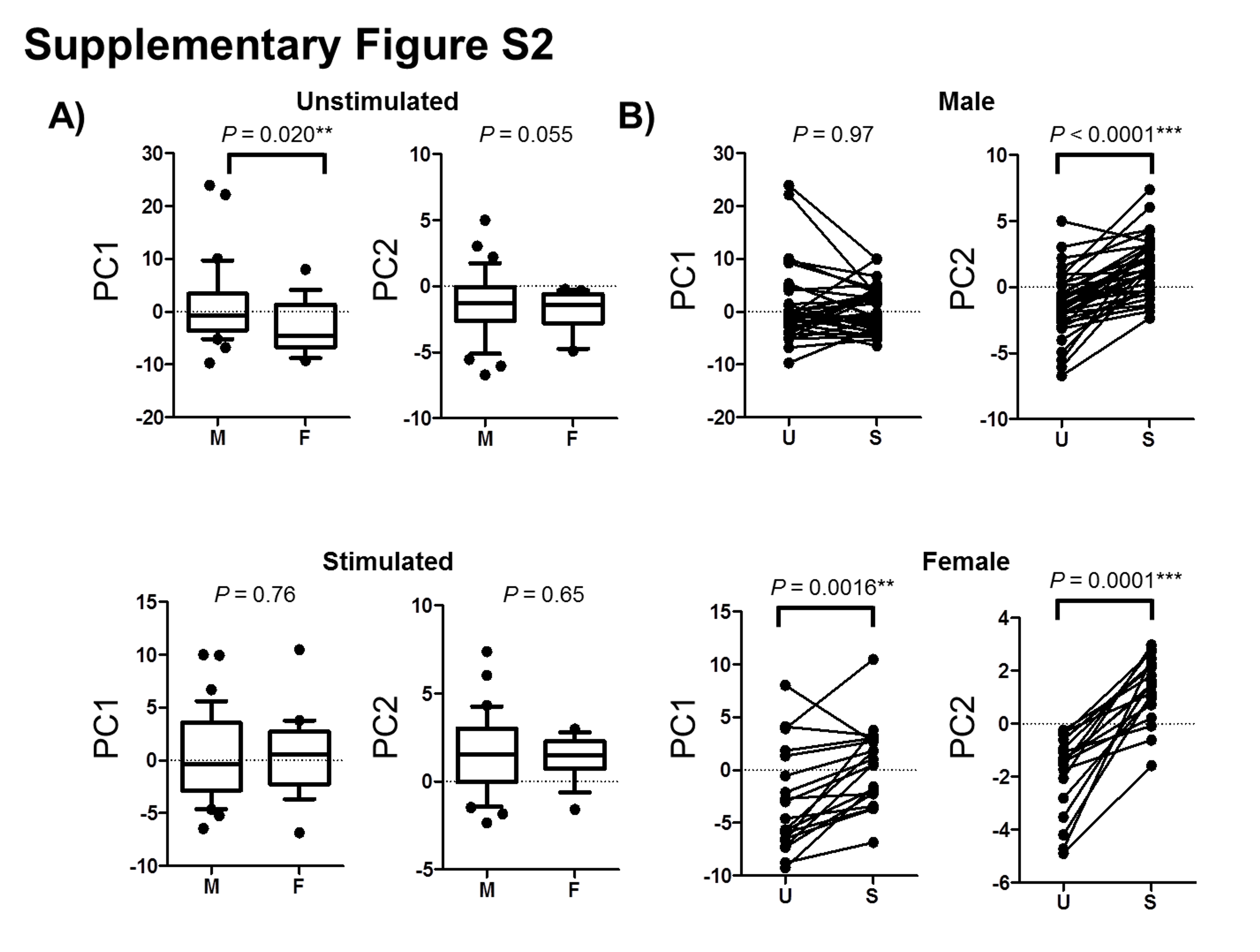

Supplement: S2 Fig — Comparisons between (A) sex, and (B) sex for unstimulated and stimulated saliva groups. P-values were calculated using the (A) Mann-Whitney test and Wilcoxon matched pairs test (B), respectively. (TIF) [file pone.0183109.s002.tif]

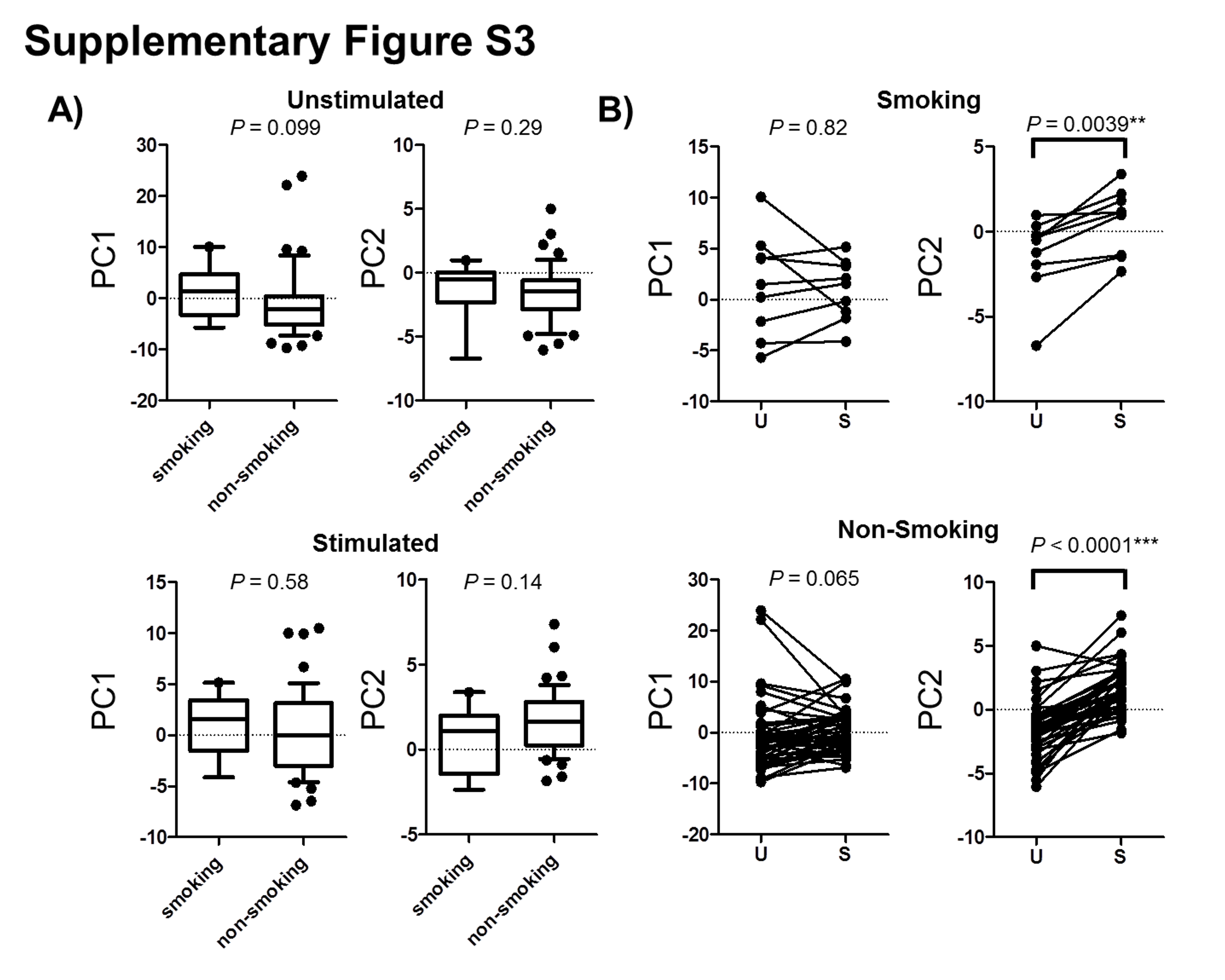

Supplement: S3 Fig — Comparisons between (A) smoking and non-smoking, and (B) comparison of unstimulated and stimulated saliva among subjects with each smoking habit. P-values were calculated using A) the Mann-Whitney test, and B) Wilcoxon matched pairs test. (TIF) [file pone.0183109.s003.tif]
